# Key principles of the KiDD (kids’ development diagnosis and determining the risk of autism for children from 1.5 to 6 years) methodology development and comparison of results with other methods

**DOI:** 10.1017/gmh.2024.85

**Published:** 2024-10-22

**Authors:** Olena Iniutina

**Affiliations:** PhD in Psychology, Taras Shevchenko National University of Kyiv, Kyiv, Ukraine

**Keywords:** likelihood of ASD (Autism Spectrum Disorder), testing children with ASD, testing developmental delay in children, KiDD (Kids’ Development Diagnosis and Determining the Risk of Autism), psychological diagnostics of skills for children with autism

## Abstract

The author outlines the basic principles of creating the KiDD methodology (Kids’ Development Diagnosis and Determining the Risk of Autism) for children aged 1.5 to 6 years old in the form of a mobile application. Users of the KiDD (parents or specialists) instantly receive information about the general development of the child in comparison with the age at which certain skills emerge. This includes information about the developmental age in months for each developmental area (speech and communication, socialization and behavior, cognitive skills, physical development and self-care), the developmental age for each specific skill of the child (up to 100 skills in each age category from 1.5 to 6 years) and the likelihood of autism. Additionally, users receive an automatically generated Individual Development Plan, consisting of skills that follow those that the child already has. The author provides statistical data comparing the results obtained through the KiDD with the results of widely accepted tests for assessing a child’s developmental level and the likelihood of autism. The article presents comparative data of the results of 199 participants using the KiDD along with their respective diagnoses and results obtained through testing provided by psychologists and remote assessment provided by parents.

## Impact statement

The simple and accessible testing of child development for ages 1.5–6 in the application, based on the understandable concept of “child’s skills” for parents, will allow them to identify a list of skills (with the indication of the psychological age of development for each) that are delayed in development and the degree of the likelihood of autism (low, moderate, medium or high). Instant receiving of the test result and the Individual Child Development Plan (with skills following those that are already present in the child), in our opinion, significantly increases the possibility of early intervention. The stages of method development and scientific justification were described by the author in the previous publications and can be found via Open Researcher and Contributor ID (ORCID) 0009-0003-9492-0915. The methodology is the subject of author’s PhD dissertation which was defended in August 2024.

## Introduction

Following the Diagnostic and Statistical Manual of Mental Disorders, Fifth Edition (DSM-5), “ASD encompasses pervasive impairments in social communication and interaction, along with restricted, repetitive patterns of behavior, interests, or activities that significantly impact daily functioning. “Signs of autism are observed from early childhood. Autism persists throughout life and has an impact on the child’s behavior and personality. However, according to numerous studies, early intervention can equip children with skills that significantly improve their quality of life and facilitate adaptation (Zwaigenbaum et al., 2015).

Research has demonstrated that with early intervention 76.5% of autistic children subsequently have the opportunity to learn within a mainstream school program. In other words, the majority of children achieve a level of socialization necessary for a comfortable life in society (Magiati et al., 2011; Karanth and Chandhok, 2013; Smith et al., 2021).

Wars, epidemics, long-term quarantine, financial constraints and the unavailability of psychological support due to remote living and the financial condition of families have deprived many children of the chance to receive well-timed and accessible qualified psychological diagnosis and assistance. One of the ways to overcome this problem has been the rapid dissemination and use of distance technologies in conducting developmental assessments of children (Ashburner et al., 2016).

According to the review of ten studies conducted by Stavropoulos et al. (2022), the results of ASD diagnosis using remote methods coincide with traditional diagnosis results in 80–91% of cases, confirming the expediency of applying mobile applications for detecting autism in young children. A review of 19 autism screening tests made by Gharamaleki et al. (2022) shows that among these tests, 63.1% are completed as a questionnaire by parents because parents know more about their children’s emotions and feelings. Only a few screening tests are available in electronic format for parents. As a result of using these tests, the user receives information about a certain degree of the likelihood of autism without identifying the specific skills that should be trained.

To formulate a detailed development program, psychologists use 3–5 tests for diagnosing each child (Randall et al., 2018). We did not find any available testing for parents and psychologists for children aged 1 to 6 that combines the assessment of general child development and ASD manifestations at the same time (with the determination of the development level in months for each specific skill and highlighting skills that are delayed in autism) and automatically generated Individual Development Plan. This is because a child’s development can be uneven (Joseph et al., 2002).

A key finding from the survey of 1,047 parents about their experiences of getting an autism diagnosis for their children in the United Kingdom was that parents typically encounter a delay of 3.5 years between first contacting a healthcare professional and receiving a formal diagnosis of ASD for their child. Interestingly, according to the survey, parents are the first to notice the signs of autism (Crane et al., 2016). Such studies have not been performed in Ukraine.

Involving parents in the process of assessment of a child’s skills, provided with a specific methodological framework, will help to increase the percentage of early detection of developmental delays and the likelihood of autism, and it will contribute to the child’s development (Oono et al., 2013).

This situation requires bringing psychological assessment closer to parents and children and creating effective distance methods capable of efficiently detecting developmental delays and the likelihood of autism from the second year of the child’s life. The methodology KiDD can be used by psychologists or by parents independently.

The article describes the basic principles of creating the KiDD (Kids’ Development Diagnosis and Determining the Risk of Autism) methodology for ages 1.5–6 in the form of a mobile application with automatic result calculation and the creation of an Individual Child Development Plan. Additionally, data are provided by comparing the results of the KiDD methodology with Kiphard (Zinnhuber) tests for overall development and Modified Checklist for Autism in Toddlers (M-CHAT) and Autism Treatment Evaluation Checklist (ATEC) for the likelihood of autism, along with diagnoses of research participants. For comparison, we selected the abovementioned methodologies that have proven to be supportive tools for the assessment of children on the spectrum (Adamu and Abdullahi, 2022). The tests are widely used worldwide and standardized and free in Ukraine in electronic format. The use of these methodologies is not only permitted by specialists but also by parents, which was a key requirement in selecting methodologies for comparison.

The methodology KiDD can be used by psychologists, tutors, educators, teachers and social workers in children’s hospitals, kindergartens, rehabilitation centers and schools. In addition, free use of home assessment by parents could be provided even before the official clinical diagnosis is established. The mentioned testing is not a diagnosis and does not replace a doctor’s diagnosis but helps parents to explain in detail to the doctor which skills are missing in the child.

Psychologists and parents will receive a detailed psycho-educational profile of the child as a result of the diagnosis, and the automatically generated Individual Development Plan will provide a complete list of skills that need to be developed in the child, starting from those that are delayed the most to those that are delayed less. The outlined plan corresponds to the psychological development of the child rather than his or her physical age. Currently, the application is undergoing final testing and will be available to users in 2024 (copyright registration of the methodology on February 10, 2023, under number c20230074 in the Ukrainian National Office of Intellectual Property and Innovation).

The aim of the KiDD methodology is to provide a convenient and effective tool for the detailed assessment of children’s skills. The author does not aim to bring an autistic child to age-appropriate norms. Instead, as a result of the testing, users receive a list of skills beyond those already possessed by the child, to assist in their development and provide the child with more opportunities in life.

## Methods

The goal and objectives of the research determined the use of the theoretical-analytical method of psychological research; clinical-psychological method; psychodiagnostic method (Kiphard, Zinnhuber, M-CHAT and ATEK tests), methods of statistical processing (Spearman’s method, contingency tables, expert assessment method (Delphi method) and descriptive statistics).

To achieve the research goal, a combination of theoretical methods was applied in the first stage, including the analysis of psycho-pedagogical literature on the issues of diagnosing development and the likelihood of autism in young children based on such criteria: psychological spheres investigated with the help of a particular methodology, the time required for its implementation, accessibility of the methodology for using by professionals and parents, the necessity for users to undergo specialized training, the availability of stimulus material and the practical usability of test results for creating an Individual Development Plan.

The analyzed methodologies assessing general development include the Denver Screening Test (Frankenburg and Dodds, 1967), Kiphard Method (2006) (Schilling and Kiphard, 1974), Zinnhuber Method (2010) (Sinnhuber, 2014), Petersi M. “Little Steps” Method (2008) (Pieterse and Treloar, 1981), Sundberg Method, Mark L. VB-Mapp (Sunberg, 2008), 100 Skills by Catherine Maurice (1996) (Maurice, 1994) and general developmental norms for children in the 2022 version (Zubler et al., 2022).

The methodologies assessing the likelihood of autism include a screening questionnaire, the M-CHAT (Robins et al., 2009), the Childhood Autism Rating Scale (CARS) (Schopler et al., 1980), Autism Diagnostic Interview-Revised (ADI-R) (Rutter et al., 2003), Autism Diagnostic Observation Schedule (ADOS) (McCrimmon and Rostad, 2014), Developmental Profile Scale for determining the psycho-educational profile of children with ASD (PEP-R) (Reichler et al., 1990) and the Autism Treatment Evaluation Checklist (ATEC) (Edelson and Rimland 1999). The review of methodologies was conducted in previous publications by the author.

In the second stage, to create the methodology KiDD, the presence and nature of the main 620 skills reflecting the general development of children aged 1.5 to 6 years in four development areas were identified. These skills were categorized into seven age groups (1.5, 2, 2.5, 3, 4, 5 and 6 years) with up to 100 skills in each age category. Additionally, skills, the delay of which indicates the likelihood of autism (“autism signs”), were identified for each age category. All skills from different age categories were combined into a “hierarchy of skills,” where each skill in the younger age category has its version in older age categories from 1.5 to 6 years.

To assess the presence of these skills, stimulus materials were created (colorful diagnostic tasks for children on 163 pages), and their use was tested in person with a sample of 79 children.

This approach provided the opportunity to identify the nature of existing skills in neuro typical children (comparison group) and children with different levels of developmental delay and the likelihood of autism (neurodivergent children).

As a result, the diagnostic method for assessing the development of children’s skills (hereinafter referred to as the KiDD test) was created. It is the first test that simultaneously diagnoses general development in main areas (relative to age norms) and the likelihood of autism, providing subsequent questions based on previous answers. The combination of assessing general development and the likelihood of autism in one test is justified by the fact that ASD is a spectrum of disorders where a delay in up to half of all a child’s skills may occur.

**Figure 1.**
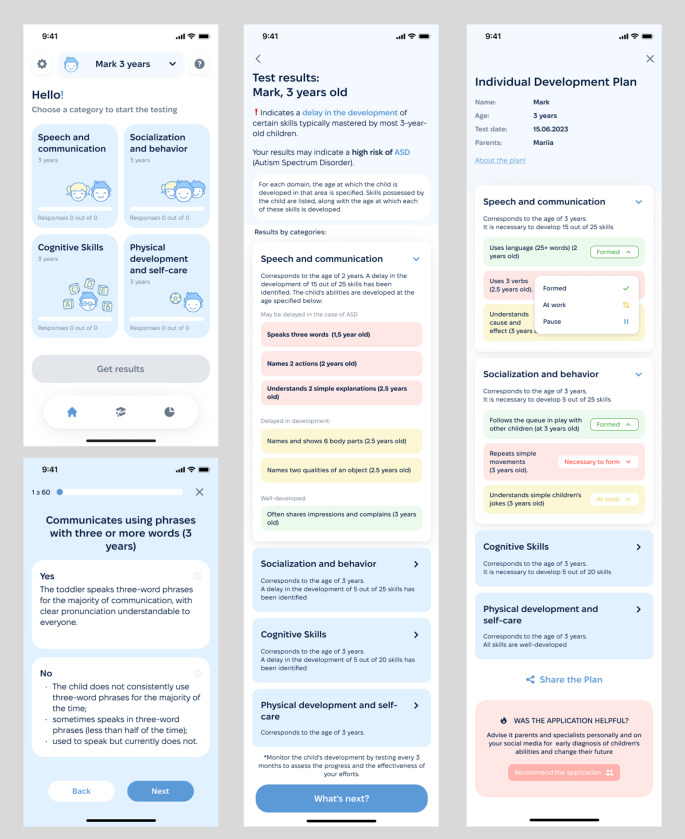
Examples of screens from the application created according to the KiDD methodology.

Figure 1 shows examples of the application screens.

Screen 1 displays the developmental spheres being diagnosed; Screen 2 shows a sample test question assessing a specific skill; Screen 3 illustrates a sample test result; Screen 4 provides an example of an Individual Development Plan.

In the third stage, statistical analysis methods were applied to confirm the effectiveness of the KiDD methodology by comparing it with other widely accepted tests and children’s diagnoses. The data collected during the study were analyzed using statistical methods, including nonparametric analysis (Spearman’s method). The process of data processing involved their collection, correction and systematization, which were performed using Microsoft Office Excel 2016 electronic spreadsheets. To visualize and conduct a more detailed analysis of the obtained results, the Jamovi v. 2.3.18 program (20) was used (Şahin and Aybek, 2019).

Spearman’s method was applied to determine the relationships between the KiDD methodology and other methods, as well as children’s diagnoses. Contingency tables were used for certain nominal indicators, with the strength of the relationship being measured using the Phi coefficient. Overall descriptive indicators are presented in percentage ratios.

Standardization, validation and testing were conducted on a representative sample of 199 Ukrainian children aged 18–72 months. A psychologist tested 100 children using the KiDD methodology, while parents tested 99 children remotely using the application. The results for each of the two groups are presented separately, and the results of testing for both groups were compared.

## Results and discussion

Testing according to the KiDD methodology lasted for more than 3 years, from 2021 to 2024. Standardization, validation and testing were conducted on a representative sample of 199 Ukrainian children aged 18–72 months. A psychologist tested 100 children using the KiDD methodology, while parents tested 99 children remotely using the KiDD methodology in the application.

Therefore, the effectiveness of the KiDD methodology was assessed through a comparison with other widely accepted tests to determine convergent and discriminant validity; a comparison with children’s diagnoses to establish criterion validity regarding overall development and risk of disorders; a comparison of results from face-to-face, a remote and self-diagnosis; and comparison with results of repeated testing over time to determine test–retest reliability and prognostic validity regarding autism spectrum disorders and developmental delay. Content and construct validity was assessed by 14 experts using the Delphi method.

To compare the effectiveness of the KiDD methodology, all 100 participants tested by a psychologist also underwent testing using the Kiphard (18–48 months) and Zinnhuber (49–72 months) methods for general development and the M-CHAT (18–30 months) and ATEC (31–72 months) methods for the likelihood of ASD. Among children with developmental disorders, each of the four developmental areas was evaluated separately, as autism often involves uneven development, making it impossible to calculate the average development of all four spheres and the overall psychological age of the child.

For the age group of 18–36 months, the comparison of the KiDD methodology with the Kiphard (18–48 months) and Zinnhuber (49–72 months) methodologies was considered positive if the results for a particular area did not differ by more than 3 months, and for the age group of 37–72 months, the comparison results were considered positive if they differed by no more than 6 months.

The likelihood of autism (or absence of it) according to the KiDD method was compared with the risk of ASD based on the results of the M-CHAT (18–30 months) and ATEC (31–72 months) tests and the child’s diagnosis.

For the M-CHAT method (18–30 months), the comparison result was considered positive if both tests showed the likelihood of autism (or absence of it).

For the ATEC method, the comparison result was considered positive if the KiDD test showed the likelihood of autism, and the ATEC test showed any degree of autism manifestation except low. The degree of risk was not compared because ATEC diagnoses the degree of autism manifestation (thus, any level of the likelihood of autism, except low, implies the presence of a risk), while the KiDD test determines the degree of the likelihood of autism (not the degree of its manifestation).

See, which tests the KiDD methodology was compared with, in Table 1.

**Table 1.** Comparison of KiDD test with other methods for general development

#	The areas of child development of the test being compared	The areas of child development of the tests compared with KiDD for general development			
	KiDD	Kiphard (18–48 months)	Zinnhuber (49–72 months)	M-CHAT (18–30 months)	ATEC (31–72 months)
1	Speech and communication	Auditory Perception Speech	Auditory Perception Speech	–	Communication skills
2	Socialization and behavior	–	–	Socialization	Socialization
3	Cognitive skills	Visual perception	Visual perception	–	Sensory skills Cognitive abilities
4	Physical development and self–care	Fine motor skills Gross motor skills	Fine motor skills Gross motor skills	–	Health Physical development Behavior

When comparing the KiDD test results of 49 neurodivergent children (comparison group) according to their health condition (absence of diagnosis), 96% of the neurodivergent children obtained the corresponding results in the group tested by a psychologist (19 children), and 90% of the children in the group tested remotely by parents using the application (30 children).

The comparison results of the KiDD test with Kiphard (Zinnhuber) tests (general development) and M-CHAT/ATEC tests (likelihood of autism) in a sample of 100 children tested by a psychologist were as follows: 93% correspondence in the “Speech and Communication” domain (Spearman’s rho coefficient = 0.966, df = 98, p < 0.001); 90% correspondence in the “Cognitive Development” domain (Spearman’s rho coefficient = 0.959, df = 98, p < 0.001); 93% correspondence in the “Physical Development and Self-care” domain (Spearman’s rho coefficient = 0.969, df = 98, p < 0.001); 77% correspondence in the “Socialization and Behavior” domain ( 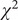 value = 25.8, df = 1, p < 0.001). The obtained value ( 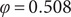 ) indicates a moderately strong positive correlation.

The ability of the KiDD test to identify the likelihood of autism is 84%, which indicates the high statistical significance of the results( 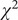 value = 44.4, df = 1, p < 0.001) and a moderately strong correlation between the results (correlation strength 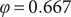 ). See the results of the comparison in Table 2.

**Table 2.** The percentage of correspondence between the KiDD test and the Kiphard (18–48 months) and Zinnhuber (49–72 months) tests for general development and with M-CHAT (18–30 months) and ATEC (31–72 months) for the likelihood of autism of 100 children tested by a psychologist

#	Developmental areas	Percentage of correspondence for general development	Percentage of correspondence for likelihood of autism
1	Speech and communication	93%	84%
2	Socialization and behavior	77%	
3	Cognitive skills	90%	
4	Physical development and self–care	93%	

The correspondence of the KiDD test results to the diagnosis regarding overall development is 98%. The correspondence of the KiDD test results to a child’s diagnosis of ASD is 73%. The result of the comparative analysis regarding the correspondence of the KiDD method results to an ASD diagnosis indicates the high statistical significance of the results, emphasizing the differences between the KiDD method results and the actual ASD diagnosis ( 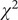 test value: 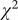 = 28.2, df = 1, p < 0.001). The Phi coefficient ( 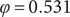 ) indicates a moderately strong correlation between the presence of an ASD diagnosis and the results of risk assessment using the KiDD method. Due to the late diagnosis of ASD, the percentage of children whose likelihood of autism according to the KiDD test matches the official diagnosis may be higher than 73% when the diagnosis is established.

When comparing the results of 99 children who were independently tested by parents using the KiDD test with their diagnoses regarding overall development (presence or absence of developmental disorders), 87% of children obtained corresponding results; namely, the KiDD test showed developmental disorder when a diagnosis of developmental disorder was present. The correlation is presented in contingency tables. The 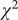 value = 57.6, df = 1, p < 0.001. The Phi coefficient was used to measure the strength of the relationship. The obtained value ( 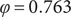 ) indicates a strong positive correlation.

When comparing the results of 99 children with their diagnosis (ASD) using the KiDD test – the presence or absence of ASD diagnosis or suspected ASD – 70% of the children obtained corresponding results; namely, the KiDD test showed ASD likelihood when an ASD diagnosis or suspected ASD was present. The correlation is presented in contingency tables. The 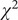 value = 31.2, df = 1, p < 0.001. The Phi coefficient was used to measure the strength of the relationship. The obtained value ( 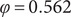 ) indicates a moderately strong positive correlation. See the results of the comparison in the Table 3.

**Table 3.** The percentage of correspondence between the KiDD test and the diagnosis of 99 children (18–72 months) tested by parents in the application for general development and for the likelihood of autism

#	Correspondence between the KiDD test and the diagnosis of children	
1	For general development	87%
2	For the likelihood of autism	70%

When comparing the results of 38 children who were tested twice with the KiDD test (with a gap of 3–12 months), conducted by a psychologist, with the results of the KiDD test conducted independently by parents regarding overall development (presence or absence of developmental disorders), in 97% of cases the results of both tests coincided – developmental delay was identified both during testing by psychologist and during testing by parents. The 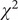 value = 33.0, df = 1, p < 0.001. The Phi coefficient was used to measure the strength of the relationship. The obtained value ( 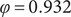 ) indicates a strong positive correlation.

When comparing the results of 38 children who were tested twice with the KiDD test, conducted by a psychologist, with the results of the KiDD test conducted independently by parents regarding ASD likelihood (presence or absence of the likelihood of autism), in 92% of cases the results of both tests coincided – ASD likelihood was identified both during testing by psychologist and during testing by parents. The 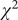 value = 24.6, df = 1, p < 0.001. The Phi coefficient was used to measure the strength of the relationship. The obtained value ( 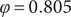 ) indicates a strong positive correlation.

The results demonstrate high test–retest reliability and prognostic validity of the KiDD test. The testing results conducted by a psychologist on a sample of 100 children were compared with the results of independent testing by parents using the application on 99 children.

During testing conducted by a psychologist, the percentage of children whose diagnosis matched the result of the KiDD test regarding overall development (presence or absence of developmental disorders) was 98%, while during independent testing of 99 children, conducted by parents, it was 90%. There is an 8% difference in favor of testing by a psychologist, which, in our opinion, is a minor difference.

During testing conducted by a psychologist, the percentage of children whose diagnosis matched the result of the KiDD test regarding the likelihood of autism (presence or absence of ASD, suspected ASD) was 73%, while during independent testing of 99 children, conducted by parents, it was 70%. There is a 3% difference in favor of testing by a psychologist, which, in our opinion, is a minor difference. See the results of the comparison in Table 4).

**Table 4.** Comparison of results of the KiDD and diagnosis of 199 children undergoing examination by a psychologist (100 children) and remotely by parents in the application (99 children)

#	Testing format	The percentage of children (18–72 months) whose diagnosis (ASD) matched the result of the KiDD regarding the likelihood of autism	Percentage of children (18–72 months) whose results are consistent with KiDD and diagnosis regarding overall development
1	By psychologist	73%	98%
2	Remotely by parents in the app	70%	90%
3	Difference	3%	8%

## Conclusion

The strengths of this research include its innovativeness, as the practical outcome resulted in the implementation of the methodology in a mobile application. A thorough analysis of the results was conducted, allowing for a better understanding of the essence and potential limitations of the methodology. A comparative analysis with four widely accepted tests and diagnoses was carried out, enabling the determination of the effectiveness of the new methodology compared to standard approaches. A detailed investigation regarding various aspects of the validity and reliability of the methodology was performed.

The weaknesses of the methodology include the sample size of 199 participants, which is planned to be increased in future studies. The limitations of the methodology may include the monocultural nature of the sample. Since developmental norms and autism signs do not significantly differ across countries, the methodology can be used for assessing the skills of children from various nationalities with some adaptation.

Based on this research, it can be concluded that the KiDD test in the form of a mobile application could become a useful and effective tool for determining the level of overall development and the likelihood of autism, both in face-to-face and remote formats of psychological assessment conducted by psychologists and by parents. Further research is required to explore the use of the KiDD methodology for testing children of other nationalities in English, and further independent use of the methodology by parents in the form of a mobile application.

## Data Availability

For access to materials, please contact the author at olenainiutina@gmail.com.
