# Supplementary material for: Key principles of the KiDD (kids’ development diagnosis and determining the risk of autism for children from 1.5 to 6 years) methodology development and comparison of results with other methods
Source: Glob Ment Health (Camb). 2024 Oct 22;11:e88. doi: 10.1017/gmh.2024.85 (PMC11504923; doi:10.1017/gmh.2024.85)
Supplement: Peer review history

# Editor report: Recommendation: Key principles of the KiDD (kids’ development diagnosis and determining the risk of autism for children from 1.5 to 6 years) methodology development and comparison of results with other methods — R0/PR2

**DOI:** 10.1017/gmh.2024.85.pr2

**Affiliations:** Psychiatry, Makerere University CHS, Uganda

In the discussion, please make a statement about cultural adaptability of KiDDS as this is a potentially important tool for early screening of autism in LMIC with few trained personel in this area.

# Editor report: Decision: Key principles of the KiDD (kids’ development diagnosis and determining the risk of autism for children from 1.5 to 6 years) methodology development and comparison of results with other methods — R0/PR3

**DOI:** 10.1017/gmh.2024.85.pr3

**Affiliations:** Johns Hopkins University Bloomberg School of Public Health, United States

No accompanying comment.

# Editor report: Recommendation: Key principles of the KiDD (kids’ development diagnosis and determining the risk of autism for children from 1.5 to 6 years) methodology development and comparison of results with other methods — R1/PR5

**DOI:** 10.1017/gmh.2024.85.pr5

**Affiliations:** Psychiatry, Makerere University CHS, Uganda

Dear Author,

Please respond to the reviewer comments making sure that local, regional and global literature is appropriately well covered.

# Editor report: Decision: Key principles of the KiDD (kids’ development diagnosis and determining the risk of autism for children from 1.5 to 6 years) methodology development and comparison of results with other methods — R1/PR6

**DOI:** 10.1017/gmh.2024.85.pr6

**Affiliations:** Johns Hopkins University Bloomberg School of Public Health, United States

No accompanying comment.

# Editor report: Recommendation: Key principles of the KiDD (kids’ development diagnosis and determining the risk of autism for children from 1.5 to 6 years) methodology development and comparison of results with other methods — R2/PR8

**DOI:** 10.1017/gmh.2024.85.pr8

**Affiliations:** Psychiatry, Makerere University CHS, Uganda

No accompanying comment.

# Editor report: Decision: Key principles of the KiDD (kids’ development diagnosis and determining the risk of autism for children from 1.5 to 6 years) methodology development and comparison of results with other methods — R2/PR9

**DOI:** 10.1017/gmh.2024.85.pr9

**Affiliations:** Johns Hopkins University Bloomberg School of Public Health, United States

No accompanying comment.
